# Comprehensive ecosystem analysis of two small, urban wetlands from Costa Rica

**DOI:** 10.3897/BDJ.13.e154073

**Published:** 2025-08-01

**Authors:** Viviana Arguedas, Marco D. Barquero

**Affiliations:** 1 Carrera de Turismo Ecológico, Recinto de Paraíso, Universidad de Costa Rica, Cartago, Costa Rica Carrera de Turismo Ecológico, Recinto de Paraíso, Universidad de Costa Rica Cartago Costa Rica; 2 Carrera de Turismo Ecológico, Recinto de Grecia, Sede de Occidente, Universidad de Costa Rica, Alajuela, Costa Rica Carrera de Turismo Ecológico, Recinto de Grecia, Sede de Occidente, Universidad de Costa Rica Alajuela Costa Rica; 3 Sede del Caribe, Universidad de Costa Rica, Limón, Costa Rica Sede del Caribe, Universidad de Costa Rica Limón Costa Rica

**Keywords:** biodiversity, flora, fauna, Paraíso, water quality

## Abstract

Urban wetlands, although important ecosystems for biodiversity, are highly vulnerable due to human activities. This work aims to study the water quality and the biodiversity present in two small, urban wetlands in Costa Rica, comparing the information collected with that reported for similar wetlands across the Neotropics. We performed a thorough literature review and field visits to elaborate a species list of terrestrial vertebrates and plants. We also analysed water samples from both wetlands to determine the water quality of the study sites. We also assembled a species list extracted from literature referring to urban wetlands. We identified a total of 453 species from our study sites (160 plants and 293 animals), with a low percentage (41%) of shared species between both sites. Fourteen species are considered threatened and two species are endemic to Costa Rica. We found low similarity amongst urban wetlands across the Neotropics. The water from both wetlands showed signs of contamination, such that they are considered for conservation of nature. We suggest that the conservation of urban wetlands should be part of the policies of local governments and more efforts for environmental education should be carried out to protect this type of ecosystem.

## Introduction

Wetlands are ecosystems that provide multiple services to humans, including natural products (e.g. fish and wood), carbon sequestration, protection from flooding, water quality improvement, mitigation of shoreline erosion and areas for recreation and tourism (Bhowmik 2020). Wetlands host particular ecological communities composed of both terrestrial and aquatic organisms and differences in biodiversity amongst wetlands depend on the specific characteristics of each wetland, such as hydrological, geomorphological and edaphic characteristics, as well as water chemistry and climatic conditions (Gopal 2009).

The Ramsar Convention, signed in 1971, established a global network of wetlands of international importance that promotes the conservation and sustainable use of these ecosystems (Gell et al. 2023). However, the effectiveness of the Ramsar Site Network can be improved by increasing the number of sites and wetland area, increasing the functional, geographical and biological representativeness and performing effective management of these ecosystems (Finlayson et al. 2011, Kingsford et al. 2021). In this sense, even small wetlands can make a substantial contribution to preserving species richness, metapopulation dynamics and overall functionality of wetland area (Gibbs 1993, Biggs et al. 2017).

Regardless of their size, wetlands face several threats and challenges for their permanence due to direct or indirect human activities. For example, pollution, overexploitation of resources (e.g. water use, aquaculture etc.), environmental modification due to urbanisation and agriculture and climate change have negatively impacted Ramsar sites worldwide (Xu et al. 2019). In addition, it has been proposed that the loss and degradation of wetlands occurs faster in tropical regions than in temperate ones (Junk et al. 2013, Mitsch and Hernandez 2013). The vulnerability of wetlands has been studied in several countries across the Americas, such as USA, Mexico, Argentina and Chile (Mitsch and Hernandez 2013, Ravit et al. 2017, Dunmeyer 2018, Raimondo et al. 2019, Rojas et al. 2019). Small, urban wetlands are considered highly vulnerable because they survive within a matrix of altered environments and, in many cases, are still perceived as expendable (Semlitsch and Bodie 1998, Boyer and Polasky 2004).

In Costa Rica, several studies have analysed the importance, management and conservation status of the country’s wetlands, highlighting the importance of comprehensive management of these ecosystems (Daniels and Cumming 2008, Mitsch et al. 2008, Kaplan et al. 2011, Camacho Navarro et al. 2017, Rodríguez-Arias and Silva Benavides 2017, Calleja Apéstegui and López-Arias 2022, Veas-Ayala et al. 2023). The country has a total surface area of 51,100 km^2^ and ca. 11% (5697 km^2^) corresponds to 12 wetlands that are part of the Ramsar Site Network (Ramsar Convention Secretariat 2024). Only one of these wetlands of international importance (Turberas de Talamanca) is in the highlands, whereas the remaining 11 wetlands are located below 600 m a.s.l. and six of these are from coastal areas (Ramsar Convention Secretariat 2024). Additionally, an inventory of Costa Rican wetlands determined that over 75% are palustrine, although only a very small proportion (0.46%) are in the mountain ranges and the Central Valley of the country (Veas Ayala et al. 2018). However, urban wetlands of the country are less known compared to those in natural environments (Rodríguez-Arias and Silva Benavides 2017, Alonso Mezquita 2022).

The geological formation of Costa Rica, along with most parts of Panama and Nicaragua, represented the emergence of a land bridge that allowed the crossing of many taxa from South America towards North America and vice versa (Cody et al. 2010). Many species that crossed the land bridge remained in Central America and speciation produced a high biodiversity with affinities to both major land masses (Solís et al. 2024). Such North American and South American affinities are observed in the species inhabiting all ecosystems of the country, even those within urban and agricultural areas. The Central Valley of Costa Rica is a plateau at the centre of the country and includes the most populated and urbanised region, the Greater Metropolitan Area (GAM, for its Spanish acronym), with a population of more than 2 million people (INEC 2012). Pollution is a major environmental issue in the GAM and wetlands are some of the most affected ecosystems (Calvo Brenes and Mora Molina 2012).

Here, we ask the following questions: 1) how high is the biodiversity of small, urban wetlands? 2) how is the water quality of these wetlands? and 3) how is the species richness found in the studied wetlands related to that of similar ecosystems in the Neotropical Region? To answer these questions, we studied two lentic wetlands located in Paraíso, Cartago Province, an area in the Central Valley of Costa Rica that historically has been known to maintain small palustrine wetlands. Many of these wetlands have been drained to convert the land for agriculture and urbanisation (Suppl. material 1 [Fig. S1]) and the remaining ones are highly vulnerable. We intend to determine the ecosystem services and value of these small, urban wetlands in terms of the flora and fauna present, as well as the characteristics of the water. In addition, we will compare the biodiversity found at our study sites with that reported in literature for countries across the Neotropics. This information will allow us to provide suggestions for properly managing and conserving these specific wetlands, measures that could be applied regionally for ecosystems with similar conditions.

## Material and methods

### Study sites

The two wetlands used as study sites are in the canton of Paraíso, Cartago Province, Costa Rica (9°49' N, 85°52' W) and both are at an approximate elevation of 1325 m a.s.l. One wetland is within the campus of the University of Costa Rica in Paraíso called Recinto Dr. Rafael Ángel Calderón Guardia (hereafter referred to as Recinto Paraíso) and it is about 1.15 km away from the second wetland called Parque La Expresión Laguna de Doña Ana Cleto (hereafter referred to as Laguna Doña Ana), which is a public park managed by the local municipality. Recinto Paraíso covers an area of approximately 4 ha (ca. 1 ha corresponds to the wetland), whereas Laguna Doña Ana covers an area of approximately 9 hectares (ca. 1.5 ha corresponds to the wetland). The two main districts of the canton (i.e. Paraíso and Llanos de Santa Lucía), where both wetlands are located, have a combined population of ca. 41,000 people (INEC 2012) and the Púcares River drains the area (33.6 km^2^). The climate is characterised by a relatively long dry season from December to April, an average annual rainfall of 1400 mm and an average of 24.4°C of annual maximum temperature (Municipalidad de Paraíso 2021).

### Literature review and general sampling procedure

We first made a thorough revision of all documents available referring to water quality and the biodiversity occurring in the study sites. For Recinto Paraíso, we only found one study about birds (Acosta-Chaves and Ramírez-Calvo 2020) and one about amphibians (Acosta Chaves and Aguilar García 2020). For Laguna Doña Ana, we found two studies reporting a preliminary species list and water quality (Román Heracleo 2020, Alonso Mezquita 2022) and one study reporting species interactions (Acosta-Chaves and Jiménez 2016). We also collected observations of species from eBird (2024) and anecdotal sightings from personnel from both sites. The information about biodiversity extracted from these documents allowed us to prepare a preliminary species list for both flora and fauna. In addition, we carried out eight field visits to each site between 2022 and 2023, in order to record species of terrestrial vertebrates and both terrestrial and aquatic plants. The specific sampling techniques used during these visits are explained in the next sections. We visited both sites during the dry and wet seasons and, for bats and plants, we had the collaboration of experts to capture and identify species *in situ*.

### Sampling of biodiversity

For plants, we walked around the edge of each wetland and we identified all herbs, vines, shrubs and trees to the genus or species level that were up to 2 m from the border. We also identified species of shrubs and trees that were up to 15 m away from the edge. We photographed each species to have a record of their presence at each site. When *in situ* identification was not possible or reliable, we took samples or used the photographs taken to identify the species. For animals, we used different sampling techniques to observe or capture terrestrial vertebrates (i.e. amphibians, birds, mammals and reptiles). For all groups, we used Visual Encounter Surveys (Crump and Scott Jr. 1994) by walking around each study site and identifying each animal to the species level. We used this technique during both diurnal and nocturnal samplings. Amphibians and reptiles were captured by hand to obtain a reliable identification. Additionally, at each study site, we used three mistnets of 12 m long, at least 30 m apart, to capture birds early in the morning (from 05:30 h to 11:00 h) and bats at night (from 17:30 h to 21:00 h). All caught animals were photographed and released at their point of capture.

### Analysis of biodiversity

After elaborating the species list for both sites integrating our fieldwork and literature review, for each plant and animal identified to the species level, we determined their conservation status (Least Concern, Near Threatened, Vulnerable, Endangered, Critically Endangered) according to the International Union for Conservancy of Nature (IUCN) Red List of Threatened Species (IUCN 2023). We also calculated the Jaccard index to determine the degree of similarity between both sites in terms of species composition (Krebs 1999). Finally, we classified all species as native, endemic or introduced and we determined whether bird species were resident or migratory following Garrigues et al. (2023).

### Sampling of water quality

Water samples were collected by personnel of the Research Center of Environmental Contamination (CICA, for its Spanish acronym), University of Costa Rica. Two samples, one during each season (dry and wet), were collected in the wetland of each study site. The analysis of these samples included the following physical and chemical parameters: percentage of oxygen saturation, biochemical oxygen demand, ammoniacal nitrogen, organic carbon, conductivity, dissolved oxygen, nitrates, nitrites, pH, temperature, turbidity and faecal coliforms. All parameters were measured in the CICA, except for faecal coliforms that were estimated in the Laboratory of Water Microbiology, University of Costa Rica. The ranges of the parameters to determine the quality of the water are established in the Regulations for the Evaluation and Classification of the Quality of Surface Water Bodies (Decreto N°33903-MINAE-S 2007) of the Costa Rican government.

### Comparison with urban wetlands from the Neotropics

We searched the literature for studies about biodiversity in urban, freshwater wetlands and selected those that reported a species list for terrestrial vertebrates and/or plants. We found a total of 29 studies (Suppl. material 2 [Appendix S1]). For studies where part of the sampling was performed in areas that did not include a wetland or where wetlands were not located within an urban area, we only considered the species reported for urban wetlands. One study about Mexican herpetofauna was performed in a sub-estuarine wetland, but we decided to include it as we were unable to find other studies about amphibians and reptiles in urban, freshwater wetlands for that country. We extracted general information about each wetland (location, size and taxonomic group studied) and the species reported from each study. Then, we assembled separate lists for plants and animals, including the species found in our study. In the case of birds, we excluded the observations reported exclusively through eBird due to the implicit bias of including information collected by multiple people and because there is no similar resource for other taxonomic groups. We then reviewed the validity of each scientific name by using online resources for each taxonomic group as follows: Plants (WFO 2025), Amphibians (Frost 2024), Birds (eBird 2024), Mammals (Mammal Diversity Database 2024) and Reptiles (Uetz et al. 2025). We refined the species lists by removing duplicates and by excluding taxa identified to genus level, although we did retain the genera for which no other species was reported. Finally, we calculated separate Jaccard indices for plants and animals to determine the degree of similarity between wetlands across the Neotropics.

## Results

### Species occurrence

**Figure 1.**
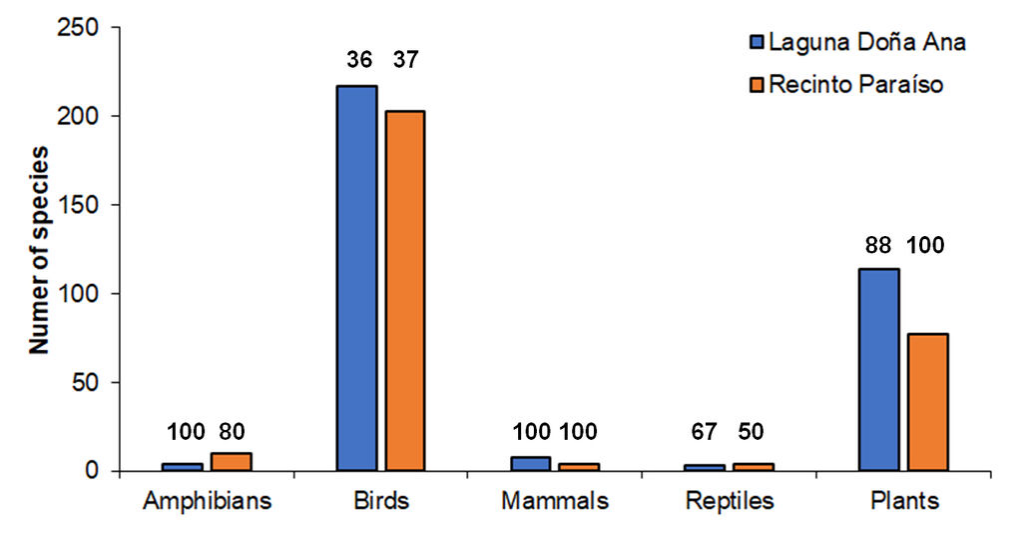
Total number of species (identified in this study and reported by the literature or personnel of each site) of terrestrial vertebrates and plants from the wetland of Laguna Doña Ana and Recinto Paraíso. The number on top of each bar represents the percentage of species observed during our fieldwork.

From our field visits, we identified a total of 100 species of plants in Laguna Doña Ana and 77 in Recinto Paraíso (Suppl. material 1 [Table S1]). The list reported here is the most comprehensive for Laguna Doña Ana and the first one for Recinto Paraíso. The literature review generated an additional 14 tree species for Laguna Doña Ana reported by Alonso Mezquita (2022) (Fig. 1). In the case of animals, we identified 232 in Laguna Doña Ana and 221 in Recinto Paraíso. The most comprehensive species list of birds for both study sites was from eBird (2024), reporting 216 species for Laguna Doña Ana and 201 for Recinto Paraíso (Fig. 1, Suppl. material 1 [Table S2]). One additional bird species (*Setophagastriata*) for Recinto Paraíso was reported by Acosta-Chaves and Ramírez-Calvo (2020). We observed 78 species in Laguna Doña Ana and 75 in Recinto Paraíso and here we report one additional species for each site (*Amazonaalbifrons* for Laguna Doña Ana and *Pirangaleucoptera* for Recinto Paraíso). For amphibians, Acosta Chaves and Aguilar García (2020) generated a list of 10 species for Recinto Paraíso and we observed eight of these. Additionally, Acosta-Chaves and Jiménez (2016) mentioned one species for Laguna Doña Ana (*Lithobatestaylori*) and here we report three additional species (Suppl. material 1 [Table S2]). We also observed two reptile species at each site, although sightings from personnel of both sites added one turtle for Laguna Doña Ana and two snakes for Recinto Paraíso (Suppl. material 1 [Table S2]). Finally, we report here the first species list of mammals for both sites, with eight species observed in Laguna Doña Ana and four in Recinto Paraíso (Fig. 1).

### Species richness and site similarity

The combined species richness of all taxonomic groups from both study sites was 453 (Table 1). Species richness was higher in Laguna Doña Ana for birds, mammals and plants, whereas Recinto Paraíso had more species of reptiles and amphibians. According to the Jaccard index, an overall similarity between both sites was 41%, although similarity was higher for birds and amphibians, relatively low for plants and mammals and no reptile species was shared for both sites (Table 1).

### Species conservation status and situation

From the total 453 species, we found that eight species are considered Near Threatened, five are Vulnerable and one is Endangered (Table 2, Suppl. material 1 [Fig. S2]). We also found that 46 species that have been introduced to the country were present at the study sites (two birds, two mammals and 42 plants). In addition, 19 species (three frogs and 16 birds) are considered to be endemic to the southern part of Central America, with only one amphibian (*Agalychnisannae*) and one bird (*Melozonecabanisi*) endemic exclusively to Costa Rica (Suppl. material 1 [Fig. S2]). Finally, we found that 85 bird species are considered migratory, 22 of which are reported exclusively for Laguna Doña Ana, 14 exclusively for Recinto Paraíso and 49 for both sites.

### Water quality

The physical, chemical and biological parameters measured from the water samples showed little variation between sites and seasons. Only the number of faecal coliforms from Recinto Paraíso and total organic carbon and conductivity from Laguna Doña Ana, all measured during the dry season, were considered outliers (Table 3). Overall, the samples from Laguna Doña Ana showed higher values than those from Recinto Paraíso (Table 3). According to the Costa Rican Regulations for the Evaluation and Classification of the Quality of Surface Water Bodies, the water from both wetlands is not for human consumption and should only be used for the conservation of aquatic communities.

### Biodiversity from urban, Neotropical wetlands

From the 29 studies obtained, we found that the wetlands sampled varied significantly in size (from 0.061 ha to 2360 ha) and no study reported a species list for both plants and all groups of terrestrial vertebrates (Suppl. material 2 [Table S4]). We extracted 501 species of fauna (50 amphibians, 384 birds, 33 mammals and 34 reptiles) and 451 species of flora. From our study, we added to this list 92 species of fauna (10 amphibians, 71 birds, 5 mammals and 6 reptiles) and 112 species of flora. Overall, we found little similarity across urban wetlands from Neotropical countries (Table 4), with the highest values found for terrestrial vertebrates and the lowest values for plants. The genera with the highest number of species reported were *Cyperus* (12 species), *Baccharis* (10) and *Solanum* (9) for plants and *Setophaga* (12 species), *Icterus* (10) and *Leptodactylus* (9) for animals (Suppl. material 2 [Fig. S3]).

## Discussion

Small, urban wetlands are important, yet fragile ecosystems that provide a variety of ecosystem services. Small lakes, ponds and swamps within a matrix of urban development are critical habitats for plants and animals, both resident and migratory. These waterbodies are commonly used by humans as recreational areas, where fishing, nature observation and relaxation are often practised. However, the lack of scientific knowledge about many of these habitats prevents us from assessing their ecological and socioeconomic value (Bhowmik 2020) and the most adequate management actions for these ecosystems to remain undamaged (Kingsford 2011). We provide scientific evidence about the importance of two small, urban wetlands of Costa Rica in terms of biodiversity present and water quality and compare our findings with those reported from similar wetlands across Neotropical countries. Our study demonstrates the uniqueness and high biodiversity present in these ecosystems, usually surviving in areas with increasing land conversion for urbanisation, cattle raising and agriculture.

In Costa Rica, the GAM is the region of the country most affected by anthropogenic activities, with natural ecosystems highly modified, degraded or even destroyed. Many wetlands have been drained, polluted or altered its species composition and Paraíso is no exception (Suppl. material 1 [Fig. S1]). Thus, plant species that should be present in the study sites are under-represented, for example, those of genera like *Baccharis*, *Eleocharis*, *Hydrocotyle*, *Ludwigia*, *Passiflora* and *Trimezia*. Such genera were reported in wetlands from other Neotropical countries (Suppl. material 2 [Fig. S3]). On the other hand, plants that have arrived via human intervention have become widespread and naturalised. This is the case of *Colocasiaesculenta* (Taro), *Megaskepasmaerythrochlamys* (Brazilian Red-cloak), *Thunbergiaalata* (Black-eyed Susan Vine) and *Zingiberspectabile* (Beehive Ginger). Additionally, these wetlands represent some of the few remaining ecosystems in the GAM for rest, food provision or even breeding sites for many animal species. This is the case for the relatively high number of migratory bird species (85 out of 267) reported for both sites.

The short distance separating both wetlands could suggest that these sites should share a great proportion of species. However, we found a low overall similarity, with even plant communities differing substantially. This could be explained because of the conditions and management observed at each site. The wetland of Laguna Doña Ana is covered by some aquatic herbs (especially *Nymphoidesindica*), its border is human-made and it is surrounded by mostly non-native plants. The local government of Paraíso is in charge of this site, offering a recreational area for local people to practise sports, observe nature and perform other activities (Alonso Mezquita 2022). Therefore, the aesthetics of Laguna Doña Ana is highly relevant and this has led to the planting of introduced trees (e.g. *Casuarina*, *Eucalyptus* and *Hesperocyparis*) or native species from other parts of the country (e.g. *Handroanthusochraceus*). On the other hand, the University of Costa Rica is in charge of Recinto Paraíso, such that the campus is orientated towards the education of students and conservation of natural resources. Thus, there are fewer introduced species and less water exposed (the area is dominated by a fern called *Cyclosorusinterruptus*) compared to Laguna Doña Ana. Additionally, the wetland retains its natural setting, surrounded by more native, local plants.

We found the same above-mentioned pattern when a larger geographic scale is considered. Urban wetlands from Neotropical countries, even those that are neighbours, hold a particular and unique species assemblage. This is reflected by the low values of the Jaccard index, suggesting that these ecosystems are not homogeneous even though they are exposed to several anthropogenic pressures (e.g. the introduction of non-native species). The site-specific biological diversity is also reflected by the number of genera, since ca. 85% of all genera of terrestrial vertebrates and ca. 90% of all genera of plants were represented by one or two species. This means that only a few genera have representatives across different Neotropical countries. We also determined that, even on these urban wetlands, Costa Rica has a higher affinity to nearby countries from North America (México) and South America (Colombia), but especially the latter. Thus, the role of the land bridge is important to explain the presence of species in the country.

The amount and quality of the water at both sites also showed some differences. In the case of Laguna Doña Ana, water remains visible even during the dry season, although desiccation is notable between both seasons. Two other studies have measured water quality from Laguna Doña Ana (Suppl. material 1 [Table S3]), with some variation across years and seasons. In this study, we detected an increase in the number of faecal coliforms during the wet season, when more birds (migratory and resident) are present. In the case of Recinto Paraíso, water is only visible during the wet season and only in areas of the wetland where *Cyclosorusinterruptus* is not present. To our knowledge, this is the first study of water quality for this wetland and only the faecal coliforms showed a very high value during the dry season, co-occurring with reparations of the sewage treatment plant. Specific sources of contamination were not identified in this study, although they are likely linked to runoff from urban and agricultural areas, as well as discharges from septic systems. This phenomenon has been documented in other places where agricultural and urban activities, characteristic of the population centres where the study sites are located, impact the quality of surface water. These activities influence physical and chemical variables and nutrient levels, both during the dry and the rainy seasons. These changes can cause degradation of the aquatic ecosystem and alter chemical and ecological processes essential for water balance (Yu et al. 2016, Mendoza et al. 2019, Arce-Villalobos et al. 2022).

The analysis of biodiversity and water quality from the two wetlands studied here and the comparison of Neotropical wetlands demonstrates the ecosystem services and the need to protect and properly manage urban wetlands. Conservation of this kind of wetlands should be of great importance for local governments. Significant efforts to study, protect and sustainably manage these ecosystems have already been carried out in some countries (e.g. Chile) (Rojas et al. 2019). As shown here, even small wetlands hold a large number of species that can disappear if such ecosystems are drained or strategies to protect them are not properly implemented (Tozer et al. 2018, Asomani-Boateng 2019). Although, in Central America, 20% of inland wetlands are protected, this region and others in Latin America also face high rates of deforestation and land conversion (Reis et al. 2017). This seems to be the case for Paraíso, where the two wetlands studied here are located, since some other small wetlands have disappeared (Calderón Vega pers. comm., Suppl. material 1 [Fig. S1]). For the wetlands at Laguna Doña Ana and Recinto Paraíso, the removal of introduced plant species and the control of potentially invasive plants is essential, especially those covering the water surface. Introduced plant species should be replaced with native plants and the water surface should be visible, allowing animals to use these areas more easily. In addition, monitoring the water quality of these wetlands and the river that feed them, will give empirical information to treat and improve water quality and the health of these ecosystems.

Finally, the involvement of local residents and visitors to the wetlands is essential so that people can have a sense of ownership, encouraging protection and sustainable use of these vulnerable ecosystems. Our findings in this research allowed the elaboration of informative material that was placed at both study sites (Suppl. material 1 [Fig. S2]), providing valuable information for the public to realise the importance of wildlife and urban wetlands. Such environmental education efforts are necessary to sensitise and raise awareness in the population about environmental issues, eliminate myths about wildlife and increase the participation of citizens in protecting natural areas.

## Supplementary Material

2A7A5A72-D142-5CBC-BCAF-7DBBD8064445 10.3897/BDJ.13.e154073.suppl1 Supplementary material 1 Online Resource 1 Data type tables, images Brief description The file contains tables of species lists of plants and terrestrial vertebrates, as well as water quality parameters. It also contains historical images of the area where the study sites are located and images of posters placed at the study sites. File: oo_1318314.docx https://binary.pensoft.net/file/1318314 Arguedas and Barquero

F7A64B00-9F1F-5D76-B151-72BAAC002CFE 10.3897/BDJ.13.e154073.suppl2 Supplementary material 2 Online Resource 2 Data type text, table, image Brief description The file contains an appendix with a list of references, a table with information about wetlands from Neotropical countries and an image about genera extracted from the literature of the appendix. File: oo_1318315.docx https://binary.pensoft.net/file/1318315 Arguedas and Barquero

## Figures and Tables

**Table 1.** Number of species (richness) and Jaccard index comparing the species composition of terrestrial vertebrates and plants from the wetland of Laguna Doña Ana and Recinto Paraíso.

**Taxonomic group**	**Species richness**			**Total**	**Jaccard index**
	**Laguna Doña Ana**	**Recinto Paraíso**	**Shared**		
Amphibians	0	6	4	10	0.40
Birds	64	50	153	267	0.57
Mammals	5	1	3	9	0.33
Reptiles	3	4	0	7	0.00
Plants	87	46	27	160	0.17
**Total**	159	107	187	453	0.41

**Table 2.** List of threatened species present at the wetland of Laguna Doña Ana and Recinto Paraíso according to the International Union for Conservation of Nature (IUCN) categories. EN = Endangered, NT = Near Threatened, VU = Vulnerable

**Taxonomic information**	**Common name**	**Site observed**	**UICN category**
*Amphibians*			
Order Anura Family Hylidae *Agalychnisannae*	Blue-sided Treefrog	Both	VU
*Birds*			
Order Apodiformes Family Apodidae *Chaeturapelagica* *Cypseloidesniger*	Chimney Swift Black Swift	Laguna Doña Ana Recinto Paraíso	VU VU
Order Passeriformes Family Icteridae *Sturnellamagna*	Eastern Meadowlark	Both	NT
Family Passerellidae *Melozonecabanisi*	Costa Rican Ground-sparrow	Recinto Paraíso	NT
Family Parulidae *Setophagastriata* *Vermivorachrysoptera*	Blackpoll Warbler Golden-winged Warbler	Recinto Paraíso Both	NT NT
Family Tyrannidae *Aphanotriccuscapitalis* *Contopuscooperi*	Tawny-chested Flycatcher Olive-sided Flycatcher	Laguna Doña Ana Both	VU NT
Order Piciformes Family Ramphastidae *Ramphastossulfuratus*	Keel-billed Toucan	Both	NT
*Mammals*			
Order Lagomorpha Family Leporidae *Oryctolaguscuniculus*	European Rabbit	Laguna Doña Ana	EN
*Plants*			
Order Arecales Family Arecaceae *Dypsislutescens*	Madagascar Areca Palm	Laguna Doña Ana	NT
Order Malpighiales Family Euphorbiaceae *Crotondecalobus*		Laguna Doña Ana	NT
Order Myrtales Family Myrtaceae *Eucalyptusdeglupta*	Rainbow Eucalyptus	Laguna Doña Ana	VU

**Table 3.** Mean ± standard deviation of physical, chemical and biological parameters measured at the wetland of Laguna Doña Ana and Recinto Paraíso during the dry and wet season, 2022.

**Parameter**	**Laguna Doña Ana**		**Recinto Paraíso**	
	**Dry**	**Wet**	**Dry**	**Wet**
Ammonium (mg/l)	0.11 ± 0.05	0.05 ± 0.02	0.07 ± 0.05	0.10 ± 0.02
Total organic carbon (mg/l)	22.93 ± 0.61	4.21 ± 0.11	2.02 ± 0.04	0.76 ± 0.03
Conductivity (µS/cm)	77.30 ± 0.48	47.85 ± 0.29	32.73 ± 0.20	34.84 ± 0.21
Biochemical oxygen demand (mg/l)	11.50 ± 0.81	3.35 ± 0.24	2.26 ± 0.16	< 1.3
Nitrates (mg/l)	< 0.25	< 0.25	< 0.25	< 0.25
Nitrites (mg/l)	0.81 ± 0.12	0.37 ± 0.02	0.011 ± 0.002	0.38 ± 0.02
Oxygen saturation (%)	41.5 ± 3.0	39.9 ± 3.0	30.6 ± 3.0	18.2 ± 3.0
Dissolved oxygen (mg/l)	3.81 ± 0.35	3.49 ± 0.35	2.84 ± 0.35	1.62 ± 0.35
pH	5.89 ± 0.24	5.93 ± 0.24	5.80 ± 0.24	5.82 ± 0.24
Temperature (°C)	23.1 ± 1.2	20.8 ± 1.2	22.2 ± 1.2	21.3 ± 1.2
Turbidity (NTU) ^a^	15.87 ± 0.87	17.20 ± 0.94	20.1 ± 1.1	9.23 ± 0.50
Faecal coliforms (MPN/100 ml) ^b^	14	220	> 1600	23

^a^ NTU = Nephelometric turbidity units (Baird et al. 2017)

^b^ MPN = Most Probable Number

**Table 4.** Coefficients of the Jaccard index for plants (upper diagonal) and terrestrial vertebrates (lower diagonal) comparing the species composition reported for urban wetlands from seven Neotropical countries

	**Argentina**	**Brazil**	**Chile**	**Colombia**	**México**	**Perú**	**Costa Rica**
**Argentina**	--	0.000	0.072	0.030	0.029	0.082	0.005
**Brazil**	0.235	--	0.000	0.005	0.000	0.015	0.000
**Chile**	0.171	0.074	--	0.027	0.036	0.044	0.005
**Colombia**	0.107	0.096	0.063	--	0.056	0.029	0.048
**México**	0.084	0.058	0.037	0.132	--	0.019	0.018
**Perú**	0.134	0.087	0.097	0.093	0.142	--	0.018
**Costa Rica**	0.051	0.067	0.041	0.217	0.125	0.075	--
