# Supplementary material for: Comprehensive ecosystem analysis of two small, urban wetlands from Costa Rica
Source: Biodivers Data J. 2025 Aug 1;13:e154073. doi: 10.3897/BDJ.13.e154073 (PMC12334924; doi:10.3897/BDJ.13.e154073)
Supplement: Supplementary material 1 — Online Resource 1 [file bdj-13-e154073-s001.docx]

Biodiversity Data Journal

**Comprehensive ecosystem analysis of two small, urban wetlands from Costa Rica**

Viviana Arguedas^‡,§^, Marco D. Barquero^|^

‡ Carrera de Turismo Ecológico, Recinto de Paraíso, Universidad de Costa Rica, Cartago, Costa Rica

§ Carrera de Turismo Ecológico, Recinto de Grecia, Sede de Occidente, Universidad de Costa Rica, Alajuela, Costa Rica

| Sede del Caribe, Universidad de Costa Rica, Limón, Costa Rica

Corresponding author: Marco D. Barquero [marco.barquero_a@ucr.ac.cr](mailto:marco.barquero_a@ucr.ac.cr)

**Table S1** List of plants identified in this study and reported by Alonso Mezquita (2022) for the wetland of Laguna Doña Ana and Recinto Paraíso. The taxonomix classification includes order, family and scientific name

| **Taxonomic classification** | **Laguna Doña Ana** | **Recinto Paraíso** |
| --- | --- | --- |
| Alismatales |  |  |
| Araceae |  |  |
| *Anthurium scandens* | X |  |
| *Colocasia esculenta* |  | X |
| *Dieffenbachia* cf. *oerstedii* |  | X |
| *Monstera deliciosa* | X | X |
| *Syngonium* sp. | X |  |
| Apiales |  |  |
| Apiaceae |  |  |
| *Cyclospermum leptophyllum* | X |  |
| *Spananthe paniculata* | X |  |
| Araliaceae |  |  |
| *Hydrocotyle umbellata* |  | X |
| Arecales |  |  |
| Arecaceae |  |  |
| *Dypsis lutescens* | X |  |
| Asparagales |  |  |
| Asparagaceae |  |  |
| *Dracaena trifasciata* | X | X |
| *Yucca gigantea* | X |  |
| Iridaceae |  |  |
| *Trimezia caerulea* |  | X |
| *Trimezia* steyermarkii |  | X |
| Orchidaceae |  |  |
| *Comparettia falcata* | X | X |
| *Epidendrum* sp. | X |  |
| *Prosthechea* sp. | X |  |
| Asterales |  |  |
| Asteraceae |  |  |
| *Ageratum conyzoides* |  | X |
| *Baccharis* sp. | X | X |
| *Bidens pilosa* | X |  |
| *Bidens reptans* |  | X |
| *Calea urticifolia* | X | X |
| *Clibadium surinamense* | X | X |
| *Conyza* sp. |  | X |
| *Crassocephalum crepidioides* | X | X |
| *Elephantopus* sp. |  | X |
| *Emilia* sp. |  | X |
| *Erechtites* sp. |  | X |
| *Hypochaeris radicata* |  | X |
| *Koanophyllon* sp. | X |  |
| *Lasianthaea fruticosa* | X |  |
| *Montanoa hibiscifolia* | X | X |
| *Sinclairia polyantha* | X |  |
| *Sonchus oleraceus* | X |  |
| *Verbesina turbacensis* |  | X |
| *Vernonanthura* sp. | X |  |
| *Youngia japonica* | X |  |
| Menyanthaceae |  |  |
| *Nymphoides indica* | X |  |
| Caryophyllales |  |  |
| Phytolaccaceae |  |  |
| *Phytolacca* sp. | X |  |
| Polygonaceae |  |  |
| *Persicaria* sp. |  | X |
| Commelinales |  |  |
| Commelinaceae |  |  |
| *Commelina* sp. | X | X |
| *Tradescantia zebrina* |  | X |
| Cucurbitales |  |  |
| Cucurbitaceae |  |  |
| *Cionosicys macranthus* | X |  |
| *Sechium* sp. | X |  |
| Cupressales |  |  |
| Cupressaceae |  |  |
| *Hesperocyparis lusitanica* ^a^ | X |  |
| Ericales |  |  |
| Primulaceae |  |  |
| *Myrsine coriacea* ^a^ | X | X |
| Fabales |  |  |
| Fabaceae |  |  |
| *Bauhinia variegata* | X |  |
| *Calliandra houstoniana var. calothyrsus* | X |  |
| *Centrosema* sp*.* |  | X |
| *Cojoba arborea* | X |  |
| *Erythrina poeppigiana* ^b^ | X |  |
| *Inga densiflora* ^b^ | X |  |
| *Inga punctata* | X |  |
| *Inga ruiziana* (aff.) | X |  |
| *Inga* sp. | X |  |
| *Inga spectabilis* ^a^ | X |  |
| *Mimosa pudica* |  | X |
| *Senna* cf. *alata* | X |  |
| *Senna papillosa* ^b^ | X |  |
| *Senna racemosa* (aff.) | X |  |
| *Senna reticulata* ^b^ | X |  |
| *Zygia longifolia* ^a^ | X | X |
| Polygalaceae |  |  |
| *Monnina* sp. | X | X |
| Fagales |  |  |
| Casuarinaceae |  |  |
| *Casuarina cunninghamiana* | X |  |
| *Casuarina equisetifolia* ^b^ | X |  |
| Gentianales |  |  |
| Rubiaceae |  |  |
| *Spermacoce* sp. | X | X |
| Lamiales |  |  |
| Acanthaceae |  |  |
| *Megaskepasma erythrochlamys* | X |  |
| *Thunbergia alata* |  | X |
| Bignoniaceae |  |  |
| *Handroanthus ochraceus* | X | X |
| *Spathodea campanulata* ^b^ | X |  |
| *Tabebuia rosea* ^a^ | X |  |
| *Tecoma stans* | X |  |
| Cordiaceae |  |  |
| *Varronia* sp. | X | X |
| Lamiaceae |  |  |
| *Hyptis capitata* |  | X |
| *Salvia* sp. |  | X |
| Orobanchaceae |  |  |
| *Castilleja arvensis* |  | X |
| Plantaginaceae |  |  |
| *Plantago major* |  | X |
| Verbenaceae |  |  |
| *Citharexylum macradenium* | X |  |
| *Lantana* sp. |  | X |
| *Stachytarpheta* sp. | X |  |
| Laurales |  |  |
| Lauraceae |  |  |
| *Persea americana* |  | X |
| *Persea caerulea* | X | X |
| Malpighiales |  |  |
| Euphorbiaceae |  |  |
| *Croton decalobus* | X |  |
| *Croton draco* | X | X |
| *Croton gossypiifolius* ^b^ | X |  |
| *Sapium glandulosum* ^b^ | X |  |
| *Sapium* sp. | X |  |
| Malpighiaceae |  |  |
| *Malpighia glabra* | X |  |
| Passifloraceae |  |  |
| *Passiflora adenopoda* | X |  |
| *Passiflora biflora* | X |  |
| Phyllanthaceae |  |  |
| *Phyllanthus* sp. | X |  |
| Malvales |  |  |
| Malvaceae |  |  |
| *Hibiscus rosa-sinensis* | X |  |
| *Malvaviscus penduliflorus* | X |  |
| *Pavonia schiedeana* | X |  |
| *Sida* sp. |  | X |
| Myrtales |  |  |
| Melastomataceae |  |  |
| *Miconia* sp. | X | X |
| *Miconia xalapensis* ^a^ | X | X |
| Myrtaceae |  |  |
| *Eucalyptus deglupta* ^a^ | X |  |
| *Eucalyptus* sp. ^a^ | X |  |
| *Psidium cattleyanum* ^a^ | X |  |
| *Melaleuca glauca* ^b^ | X |  |
| *Melaleuca viminalis* | X | X |
| *Psidium guajava* ^a^ | X | X |
| *Psidium guineense* |  | X |
| *Syzygium jambos* | X |  |
| *Syzygium malaccense* ^b^ | X |  |
| Onagraceae |  |  |
| *Ludwigia octovalvis* | X |  |
| *Ludwigia peruviana* |  | X |
| Oxalidales |  |  |
| Oxalidaceae |  |  |
| *Oxalis debilis* | X |  |
| *Oxalis* sp. | X |  |
| Piperales |  |  |
| Piperaceae |  |  |
| *Peperomia* sp. | X |  |
| *Piper* sp. | X | X |
| *Piper umbellatum* | X | X |
| Poales |  |  |
| Bromeliaceae |  |  |
| *Aechmea* sp. | X |  |
| Cyperaceae |  |  |
| *Cyperus papyrus* |  | X |
| *Cyperus* sp. | X | X |
| *Eleocharis* sp. |  | X |
| *Scleria* sp. |  | X |
| Juncaceae |  |  |
| *Juncus* cf*. effusus* |  | X |
| Poaceae |  |  |
| *Andropogon* sp. | X | X |
| *Bambusa vulgaris* ^a^ | X |  |
| *Cenchrus purpureus* |  | X |
| *Panicum* sp. | X |  |
| *Phyllostachys aurea* | X |  |
| Unidentified species 1 |  | X |
| Unidentified species 2 |  | X |
| Unidentified species 3 |  | X |
| Unidentified species 4 |  | X |
| Polypodiales |  |  |
| Thelypteridaceae |  |  |
| *Cyclosurus interruptus* |  | X |
| Ranunculales |  |  |
| Menispermaceae |  |  |
| *Cissampelos* cf. *pareira* | X |  |
| *Cissampelos* sp. |  | X |
| Rosales |  |  |
| Moraceae |  |  |
| *Ficus americana* ^b^ | X |  |
| *Ficus* cf. *citrifolia* |  | X |
| *Ficus crocata* ^b^ | X |  |
| *Ficus aurea* ^a^ | X |  |
| *Ficus* cf. *aurea* |  | X |
| *Ficus microcarpa* | X |  |
| *Ficus pertusa* ^a^ | X |  |
| *Ficus velutina* | X |  |
| Rosaceae |  |  |
| *Eriobotrya japonica* ^a^ | X |  |
| *Rubus* sp. |  | X |
| Ulmaceae |  |  |
| *Ulmus mexicana* |  | X |
| Urticaceae |  |  |
| *Cecropia obtusifolia* | X |  |
| *Cecropia* *peltata* ^b^ | X |  |
| *Cecropia* sp. |  | X |
| Santalales |  |  |
| Santalaceae |  |  |
| *Phoradendron* sp. | X |  |
| Sapindales |  |  |
| Anacardiaceae |  |  |
| *Anacardium excelsum* ^b^ | X |  |
| Meliaceae |  |  |
| *Trichilia havanensis* | X |  |
| Rutaceae |  |  |
| *Citrus x aurantiifolia* | X |  |
| Sapindaceae |  |  |
| *Cupania glabra* | X |  |
| Solanales |  |  |
| Convolvulaceae |  |  |
| *Ipomoea* sp. |  | X |
| Solanaceae |  |  |
| *Brunfelsia* sp. | X |  |
| *Cestrum* sp. | X | X |
| *Iochroma arborescens* ^a^ | X |  |
| *Solanum* cf. *americanum* |  | X |
| *Solanum* sp. |  | X |
| Zingiberales |  |  |
| Heliconiaceae |  |  |
| *Heliconia* cf. *bihai* |  | X |
| *Heliconia* sp. | X | X |
| Musaceae |  |  |
| *Musa* sp. | X |  |
| *Musa velutina* |  | X |
| Zingiberaceae |  |  |
| *Alpinia* *zerumbet* |  | X |
| *Etlingera elatior* |  | X |
| *Hedychium* sp. | X |  |
| *Zingiber spectabile* | X |  |

^a^ Species reported in this study and by Alonso Mezquita (2022).

^b^ Species reported exclusively by Alonso Mezquita (2022).

**Table S2** List of terrestrial vertebrates identified in this study and reported in the literature for the wetland of Laguna Doña Ana and Recinto Paraíso. All amphibian species for Recinto Paraíso are reported by Acosta Chaves and Aguilar García (2020), whereas species for Laguna Doña Ana are from this study. All bird species for both sites are from eBird (2024). Mammal and reptile species are from this study or anecdotal observations

| **Taxonomic classification** | **Laguna Doña Ana** | **Recinto Paraíso** |
| --- | --- | --- |
| **Amphibians** | |  |
| Anura |  |  |
| Bufonidae |  |  |
| *Rhinella horribilis* | X | X |
| Centrolenidae |  |  |
| *Hyalinobatrachium fleischmanni* |  | X |
| Craugastoridae |  |  |
| *Craugastor underwoodi* ^a^ |  | X |
| Eleutherodactylidae |  |  |
| *Diasporus diastema* ^a^ |  | X |
| Hylidae |  |  |
| *Agalychnis annae* | X | X |
| *Dendropsophus ebraccatus* | X | X |
| *Smilisca phaeota* |  | X |
| Microhylidae |  |  |
| *Hypopachus variolosus* |  | X |
| Ranidae |  |  |
| *Lithobates taylori* | X | X |
| Strabomantidae |  |  |
| *Pristimantis ridens* |  | X |
| **Birds** | |  |
| Accipitriformes |  |  |
| Accipitridae |  |  |
| *Accipiter bicolor* | X | X |
| *Buteo brachyurus* ^b,c^ | X | X |
| *Buteo jamaicensis* |  | X |
| *Buteo plagiatus* | X | X |
| *Buteo platypterus* | X | X |
| *Buteo swainsoni* |  | X |
| *Chondrohierax uncinatus* | X | X |
| *Elanoides forficatus* |  | X |
| *Elanus leucurus* ^c^ | X | X |
| *Microspizias superciliosus* |  | X |
| *Rupornis magnirostris* ^b,c^ | X | X |
| Pandionidae |  |  |
| *Pandion haliaetus* | X | X |
| Anseriformes |  |  |
| Anatidae |  |  |
| *Anas platyrhynchos* | X |  |
| *Cairina moschata* ^b^ | X | X |
| *Dendrocygna autumnalis* ^b,c^ | X | X |
| *Spatula discors* *^b^* | X |  |
| Caprimulgiformes |  |  |
| Apodidae |  |  |
| *Chaetura pelagica* | X |  |
| *Chaetura vauxi* | X | X |
| *Cypseloides niger* |  | X |
| *Panyptila cayennensis* |  | X |
| *Streptoprocne rutila* |  | X |
| *Streptoprocne zonaris* ^b^ | X | X |
| Caprimulgidae |  |  |
| *Nyctidromus albicollis* ^c^ |  | X |
| Trochilidae |  |  |
| *Amazilia tzacatl* ^b,c^ | X | X |
| *Anthracothorax prevostii* ^b^ | X | X |
| *Archilochus colubris* | X | X |
| *Campylopterus hemileucurus* | X |  |
| *Chalybura urochrysia* | X |  |
| *Chlorestes eliciae* |  | X |
| *Chlorostilbon assimilis* ^c^ |  | X |
| *Colibri cyanotus* |  | X |
| *Cynanthus canivetii* | X |  |
| *Discosura conversii* | X |  |
| *Eupherusa nigriventris* | X |  |
| *Klais guimeti* | X |  |
| *Lophornis adorabilis* | X |  |
| *Phaethornis guy* | X | X |
| *Philodice bryantae* | X | X |
| *Saucerottia hoffmanni* ^b,c^ | X | X |
| *Selasphorus scintilla* ^b,c^ | X | X |
| Cathartiformes |  |  |
| Cathartidae |  |  |
| *Cathartes aura* ^b,c^ | X | X |
| *Coragyps atratus* ^b,c^ | X | X |
| *Sarcoramphus papa* |  | X |
| Charadriiformes |  |  |
| Charadriidae |  |  |
| *Charadrius vociferus* | X | X |
| *Vanellus chilensis* ^b,c^ | X | X |
| Jacanidae |  |  |
| *Jacana spinosa* ^b,c^ | X | X |
| Recurvirostridae |  |  |
| *Himantopus mexicanus* | X |  |
| Scolopacidae |  |  |
| *Actitis macularius* | X |  |
| *Calidris mauri* | X |  |
| *Calidris melanotos* | X |  |
| *Calidris minutilla* | X |  |
| *Gallinago delicata* | X |  |
| *Tringa flavipes* | X |  |
| *Tringa melanoleuca* | X |  |
| *Tringa solitaria* ^b^ | X | X |
| Ciconiiformes |  |  |
| Ciconiidae |  |  |
| *Mycteria americana* ^b^ | X | X |
| Columbiformes |  |  |
| Columbidae |  |  |
| *Columba livia* | X | X |
| *Columbina inca* ^b^ | X | X |
| *Columbina talpacoti* ^b^ | X | X |
| *Leptotila cassinii* | X |  |
| *Leptotila verreauxi* ^b,c^ | X | X |
| *Patagioenas cayennensis* | X |  |
| *Patagioenas fasciata* |  | X |
| *Patagioenas flavirostris* ^b,c^ | X | X |
| *Patagioenas nigrirostris* | X |  |
| *Patagioenas subvinacea* | X |  |
| *Zenaida asiatica* ^b,c^ | X | X |
| Coraciiformes |  |  |
| Alcedinidae |  |  |
| *Chloroceryle amazona* | X |  |
| *Chloroceryle americana* | X |  |
| *Megaceryle torquata* | X |  |
| Momotidae |  |  |
| *Momotus lessonii* ^c^ | X | X |
| Cuculiformes |  |  |
| Cuculidae |  |  |
| *Crotophaga sulcirostris* ^c^ | X | X |
| *Piaya cayana* | X | X |
| Eurypygiformes |  |  |
| Eurypygidae |  |  |
| *Eurypyga helias* | X |  |
| Falconiformes |  |  |
| Falconidae |  |  |
| *Caracara plancus* | X | X |
| *Daptrius chimachima* | X | X |
| *Falco peregrinus* | X | X |
| *Herpetotheres cachinnans* | X | X |
| Galliformes |  |  |
| Cracidae |  |  |
| *Chamaepetes unicolor* | X |  |
| *Ortalis cinereiceps* ^c^ | X | X |
| Odontophoridae |  |  |
| *Colinus cristatus* |  | X |
| Gruiformes |  |  |
| Rallidae |  |  |
| *Aramides cajaneus* ^b^ | X |  |
| *Fulica americana* | X |  |
| *Gallinula galeata* ^b^ | X |  |
| *Laterallus albigularis* ^c^ | X | X |
| *Porphyrio martinica* ^b^ | X |  |
| Passeriformes |  |  |
| Cardinalidae |  |  |
| *Cyanoloxia cyanoides* |  | X |
| *Passerina caerulea* | X |  |
| *Passerina cyanea* | X |  |
| *Pheucticus ludovicianus* ^b,c^ | X | X |
| *Piranga bidentata* |  | X |
| *Piranga leucoptera* ^c^ | X | X |
| *Piranga olivacea* | X | X |
| *Piranga rubra* ^b,c^ | X | X |
| *Spiza americana* |  | X |
| Corvidae |  |  |
| *Psilorhinus morio* ^b,c^ | X | X |
| Fringillidae |  |  |
| *Chlorophonia elegantissima* |  | X |
| *Euphonia anneae* |  | X |
| *Euphonia hirundinacea* ^b,c^ | X | X |
| *Euphonia luteicapilla* | X | X |
| *Spinus psaltria* ^c^ | X | X |
| *Spinus xanthogastrus* | X |  |
| Furnariidae |  |  |
| *Cranioleuca erythrops* | X |  |
| *Lepidocolaptes affinis* | X |  |
| *Lepidocolaptes souleyetii* ^b,c^ | X | X |
| *Sittasomus griseicapillus* | X |  |
| *Synallaxis brachyura* |  | X |
| Hirundinidae |  |  |
| *Hirundo rustica* | X | X |
| *Petrochelidon pyrrhonota* |  | X |
| *Progne chalybea* | X | X |
| *Pygochelidon cyanoleuca* ^b,c^ | X | X |
| *Riparia riparia* | X | X |
| *Stelgidopteryx ruficollis* | X | X |
| *Stelgidopteryx serripennis* | X | X |
| Icteridae |  |  |
| *Amblycercus holosericeus* | X | X |
| *Dives dives* ^b^ | X | X |
| *Icterus galbula* ^b,c^ | X | X |
| *Icterus prosthemelas* |  | X |
| *Icterus spurius* | X |  |
| *Molothrus aeneus* | X | X |
| *Molothrus bonariensis* |  | X |
| *Molothrus oryzivorus* | X | X |
| *Psarocolius montezuma* ^b,c^ | X | X |
| *Psarocolius wagleri* | X | X |
| *Quiscalus mexicanus* ^b,c^ | X | X |
| *Sturnella magna* | X | X |
| Icteriidae |  |  |
| *Icteria virens* |  | X |
| Mimidae |  |  |
| *Mimus gilvus* ^c^ | X | X |
| Parulidae |  |  |
| *Basileuterus culicivorus* | X |  |
| *Basileuterus delattrii* ^c^ | X | X |
| *Cardellina canadensis* | X | X |
| *Cardellina pusilla* | X | X |
| *Geothlypis philadelphia* ^c^ | X | X |
| *Geothlypis poliocephala* ^c^ | X | X |
| *Geothlypis semiflava* |  | X |
| *Geothlypis tolmiei* |  | X |
| *Helmitheros vermivorum* |  | X |
| *Leiothlypis peregrina* ^b,c^ | X | X |
| *Mniotilta varia* ^b,c^ | X | X |
| *Myioborus miniatus* | X | X |
| *Myiothlypis fulvicauda* | X |  |
| *Parkesia motacilla* | X |  |
| *Parkesia noveboracensis* ^b^ | X | X |
| *Protonotaria citrea* ^b^ | X | X |
| *Setophaga* *castanea* | X | X |
| *Setophaga coronata* | X |  |
| *Setophaga dominica* |  | X |
| *Setophaga fusca* | X | X |
| *Setophaga magnolia* |  | X |
| *Setophaga pensylvanica* | X | X |
| *Setophaga petechia* ^b,c^ | X | X |
| *Setophaga pitiayumi* ^b,c^ | X | X |
| *Setophaga ruticilla* | X |  |
| *Setophaga striata* ^d^ |  | X |
| *Setophaga tigrina* | X |  |
| *Setophaga virens* | X | X |
| *Vermivora chrysoptera* | X | X |
| Passerellidae |  |  |
| *Arremon aurantiirostris* | X |  |
| *Arremonops conirostris* |  | X |
| *Atlapetes albinucha* | X | X |
| *Melozone cabanisi* ^c^ |  | X |
| *Melozone leucotis* ^b,c^ | X | X |
| *Zonotrichia capensis* ^b,c^ | X | X |
| Passeridae |  |  |
| *Passer domesticus* | X | X |
| Polioptilidae |  |  |
| *Polioptila bilineata* | X | X |
| Thamnophilidae |  |  |
| *Thamnophilus doliatus* | X | X |
| Thraupidae |  |  |
| *Coereba flaveola* ^b,c^ | X | X |
| *Dacnis venusta* |  | X |
| *Ramphocelus passerini* ^b,c^ | X | X |
| *Saltator atriceps* | X | X |
| *Saltator grandis* ^b^ | X | X |
| *Saltator maximus* ^b,c^ | X | X |
| *Saltator striatipectus* ^c^ |  | X |
| *Sporophila corvina* | X | X |
| *Sporophila funerea* |  | X |
| *Sporophila morelleti* | X | X |
| *Sporophila nigricollis* |  | X |
| *Stilpnia larvata* ^b^ | X | X |
| *Tachyphonus rufus* |  | X |
| *Tangara icterocephala* | X | X |
| *Thraupis episcopus* ^b,c^ | X | X |
| *Thraupis palmarum* ^b,c^ | X | X |
| *Tiaris olivaceus* ^b,c^ | X | X |
| *Volatinia jacarina* | X | X |
| Tityridae |  |  |
| *Pachyramphus cinnamomeus* | X |  |
| *Tityra semifasciata* | X | X |
| Troglodytidae |  |  |
| *Cantorchilus modestus* ^b,c^ | X | X |
| *Cistothorus platensis* | X | X |
| *Henicorhina leucosticta* |  | X |
| *Troglodytes aedon* ^b,c^ | X | X |
| *Troglodytes ochraceus* | X |  |
| Turdidae |  |  |
| *Catharus aurantiirostris* ^b,c^ | X | X |
| *Catharus fuscater* |  | X |
| *Catharus minimus* | X |  |
| *Catharus ustulatus* | X | X |
| *Myadestes melanops* | X |  |
| *Turdus grayi* ^b,c^ | X | X |
| Tyrannidae |  |  |
| *Aphanotriccus capitalis* | X |  |
| *Attila spadiceus* |  | X |
| *Capsiempis flaveola* | X | X |
| *Conopias albovittatus* | X |  |
| *Contopus bogotensis* ^b,c^ | X | X |
| *Contopus cooperi* | X | X |
| *Contopus lugubris* | X |  |
| *Contopus sordidulus* *^c^* | X | X |
| *Contopus virens* | X | X |
| *Elaenia chiriquensis* | X | X |
| *Elaenia flavogaster* ^b,c^ | X | X |
| *Elaenia frantzii* |  | X |
| *Empidonax albigularis* | X | X |
| *Empidonax alnorum* |  | X |
| *Empidonax flaviventris* | X | X |
| *Empidonax virescens* |  | X |
| *Legatus leucophaius* ^b^ | X | X |
| *Leptopogon superciliaris* |  | X |
| *Megarynchus pitangua* ^c^ | X | X |
| *Mionectes olivaceus* | X |  |
| *Mitrephanes phaeocercus* | X | X |
| *Myiarchus crinitus* | X |  |
| *Myiarchus tuberculifer* ^b,c^ | X | X |
| *Myiodynastes luteiventris* | X | X |
| *Myiozetetes granadensis* | X | X |
| *Myiozetetes similis* ^b,c^ | X | X |
| *Phyllomyias zeledoni* | X |  |
| *Pitangus sulphuratus* ^b,c^ | X | X |
| *Rhynchocyclus brevirostris* | X | X |
| *Sayornis nigricans* ^b,c^ | X | X |
| *Todirostrum cinereum* ^b,c^ | X | X |
| *Todirostrum nigriceps* |  | X |
| *Tolmomyias sulphurescens* ^b,c^ | X | X |
| *Tyrannus melancholicus* ^b,c^ | X | X |
| *Tyrannus tyrannus* | X |  |
| *Zimmerius parvus* ^b,c^ | X | X |
| Vireonidae |  |  |
| *Pachysylvia decurtata* | X | X |
| *Vireo flavifrons* ^b,c^ | X | X |
| *Vireo flavoviridis* | X | X |
| *Vireo leucophrys* |  | X |
| *Vireo olivaceus* | X | X |
| *Vireo philadelphicus* ^b,c^ | X | X |
| Pelecaniformes |  |  |
| Ardeidae |  |  |
| *Ardea alba* ^b^ | X | X |
| *Ardea herodias* | X | X |
| *Bubulcus ibis* *^b,c^* | X | X |
| *Butorides virescens* ^b^ | X | X |
| *Egretta caerulea* ^b^ | X | X |
| *Egretta thula* ^b,c^ | X | X |
| *Tigrisoma* *fasciatum* | X |  |
| *Tigrisoma mexicanum* | X |  |
| Threskiornithidae |  |  |
| *Mesembrinibis cayennensis* ^b^ | X |  |
| Piciformes |  |  |
| Picidae |  |  |
| *Colaptes rubiginosus* ^b,c^ | X | X |
| *Dryobates fumigatus* | X | X |
| *Melanerpes formicivorus* |  | X |
| *Melanerpes hoffmannii* ^b,c^ | X | X |
| Ramphastidae |  |  |
| *Pteroglossus torquatus* | X | X |
| *Ramphastos sulfuratus* | X | X |
| Podicipediformes |  |  |
| Podicipedidae |  |  |
| *Tachybaptus dominicus* ^b,c^ | X | X |
| Psittaciformes |  |  |
| Psittacidae |  |  |
| *Amazona albifrons* ^b^ | X |  |
| *Brotogeris jugularis* | X |  |
| *Bolborhynchus lineola* |  | X |
| *Pionus senilis* | X | X |
| *Psittacara finschi* ^b,c^ | X | X |
| *Pyrilia haematotis* | X |  |
| Suliformes |  |  |
| Anhingidae |  |  |
| *Anhinga anhinga* | X |  |
| Phalacrocoracidae |  |  |
| *Nannopterum brasilianum* | X |  |
| Strigiformes |  |  |
| Strigidae |  |  |
| *Asio clamator* |  | X |
| *Glaucidium brasilianum* | X | X |
| *Megascops choliba* ^c^ | X | X |
| Tytonidae |  |  |
| *Tyto alba* |  | X |
| **Mammals** | |  |
| Carnivora |  |  |
| Felidae |  |  |
| *Felis catus* | X |  |
| Chiroptera |  |  |
| Phyllostomidae |  |  |
| *Artibeus intermedius* |  | X |
| *Artibeus jamaicensis* | X | X |
| *Artibeus lituratus* | X |  |
| *Glossophaga soricina* | X |  |
| *Sturnira parvidens* | X | X |
| Didelphimorphia |  |  |
| Didelphidae |  |  |
| *Didelphis marsupialis* | X |  |
| Lagomorpha |  |  |
| Leporidae |  |  |
| *Oryctolagus cuniculus* | X |  |
| Rodentia |  |  |
| Sciuridae |  |  |
| *Sciurus variegatoides* | X | X |
|  | |  |
| **Reptiles** | |  |
| Squamata |  |  |
| Boidae |  |  |
| *Boa imperator* ^e^ |  | X |
| Colubridae |  |  |
| *Mastigodryas melanolomus* ^e^ |  | X |
| Dactyloidae |  |  |
| *Anolis biporcatus* |  | X |
| *Anolis laeviventris* |  | X |
| Iguanidae |  |  |
| *Iguana iguana* | X |  |
| Testudines |  |  |
| Chelydridae |  |  |
| *Chelydra acutirostris* ^e^ | X |  |
| Kinosternidae |  |  |
| *Kinosternon scorpioides* | X |  |

^a^ Amphibian species reported exclusively by Acosta Chaves and Aguilar García (2020).

^b^ Bird species observed in this study for Laguna Doña Ana.

^c^ Bird species observed in this study for Recinto Paraíso.

^d^ Bird species reported exclusively by Acosta-Chaves and Ramírez-Calvo (2020).

^e^ Reptile species reported by personnel of the study site.

**Tables S3** Physical and chemical parameters measured at the wetland of Laguna Doña Ana by three different studies. Numbers are mean ± standard deviation

| **Parameter** | **Season (Sampling year)** | | | | |
| --- | --- | --- | --- | --- | --- |
|  | **Dry (2018)** | **Wet (2018)** | **Wet (2021)** | **Dry (2022)** | **Wet (2022)** |
| Redox potential (mV) |  |  | 100,87 ± 83,09 |  |  |
| pH | 7,08 ± 1,03 | 6,66 ± 0,75 | 5,75 ± 0,18 | 5,89 ± 0,24 | 5,93 ± 0,24 |
| Dissolved oxygen (% or mg/L) ^1^ | 2,74 ± 2,51 | 1,74 ± 0,84 | 6,19 ± 7,32 | 3,81 ± 0,35 | 3,49 ± 0,35 |
| Conductivity (μS/cm) | 83,70 ± 39,47 | 64,80 ± 14,48 | 48,00 ± 6,26 | 77,30 ± 0,48 | 47,85 ± 0,29 |
| Total dissolved solids (ppm) | --- | --- | 23,96 ± 3,09 | --- | --- |
| Temperature (°C) | 22,44 ± 3,59 | 22,18 ± 2,43 | 21,01 ± 0,27 | 23,1 ± 1,2 | 20,8 ± 1,2 |
| Profundity (cm) | --- | --- | 58,01 ± 13,93 | --- | --- |
| Source | Román-Heracleo (2020) | | Alonso Mezquita (2022) | This study | |

^1^ Alonso Mezquita (2022) reports %, whereas values from Román Heracleo (2020) and this study are in mg/L.

**
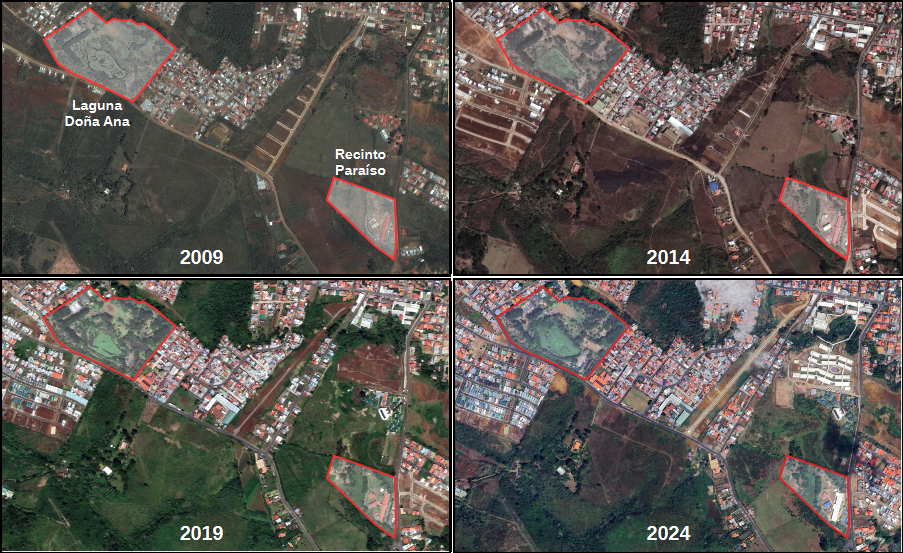
**

**Fig. S1** Landscape changes from 2009 to 2024 in the area where the studied wetlands (Laguna Doña Ana and Recinto Paraíso) are located. Images taken from Google Earth ®, with data from Maxar Technologies (images of 2009, 2014 and 2019) and Airbus (image of 2024)

**
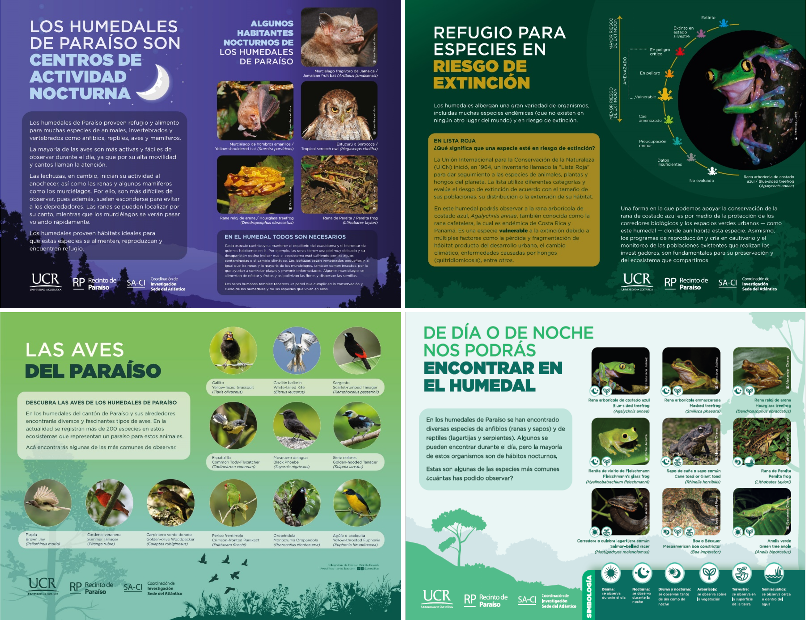
**

**Fig. S2** Posters placed at the study sites providing information about the species and the ecosystem
