# Supplementary material for: Comprehensive ecosystem analysis of two small, urban wetlands from Costa Rica
Source: Biodivers Data J. 2025 Aug 1;13:e154073. doi: 10.3897/BDJ.13.e154073 (PMC12334924; doi:10.3897/BDJ.13.e154073)
Supplement: Supplementary material 2 — Online Resource 2 [file bdj-13-e154073-s002.docx]

Biodiversity Data Journal

**Comprehensive ecosystem analysis of two small, urban wetlands from Costa Rica**

Viviana Arguedas^‡,§^, Marco D. Barquero^|^

‡ Carrera de Turismo Ecológico, Recinto de Paraíso, Universidad de Costa Rica, Cartago, Costa Rica

§ Carrera de Turismo Ecológico, Recinto de Grecia, Sede de Occidente, Universidad de Costa Rica, Alajuela, Costa Rica

| Sede del Caribe, Universidad de Costa Rica, Limón, Costa Rica

Corresponding author: Marco D. Barquero [marco.barquero_a@ucr.ac.cr](mailto:marco.barquero_a@ucr.ac.cr)

**Appendix S1** List of studies from Neotropical countries that report a species list of flora and/or fauna inhabiting urban wetlands:

1. Arana C, Salinas L (2003) Flora vascular de los Humedales de Chimbote, Perú. Revista Peruana de Biología 10 (2): 221-224.

2. Barrera-Moscoso DA, Torres-Ccasani G, Ramírez DW (2024) Primer reporte de anfibios y reptiles en el Refugio de Vida Silvestre Los Pantanos de Villa (Lima-Perú). Revista de la Academia Colombiana de Ciencias Exactas, Físicas y Naturales 48 (188): 595-605. <https://doi.org/10.18257/raccefyn.2561>

3. Cabrera Amaya DM, López Cruz JW (2019) Riqueza florística y estructura de la vegetación acuática y terrestre en el humedal El Salitre, Bogotá, Colombia. Revista de la Academia Colombiana de Ciencias Exactas, Físicas y Naturales 43 (168): 508-517. <http://dx.doi.org/10.18257/raccefyn.824>

4. Chediack SE, Ramírez-Marcial N, Martínez-Icó M, Castañeda-Ocaña HE (2018) Macrófitos de los humedales de montaña de San Cristóbal de Las Casas, Chiapas, México. Revista Mexicana de Biodiversidad 89 (3): 757-768. <https://doi.org/10.22201/ib.20078706e.2018.3.2420>

5. Cuevas JC, Íñiguez-Dávalos LI (2017) Aves del Puerto Interior Turístico Jocotepec, en el Lago de Chapala, Jalisco, México. Huitzil 18 (2): 261-271. <https://doi.org/10.28947/hrmo.2017.18.2.295>

6. Echeverry-Galvis MA, Lozano Ramírez P, Amaya-Espinel JD (2023) Long-term Christmas Bird Counts describe neotropical urban bird diversity. PLoS ONE 18 (2): e0272754. <https://doi.org/10.1371/journal.pone.0272754>

7. Fuentes Parada N (2013) Informe final: Proyecto línea base de flora y fauna del humedal de Boca Maule, comuna de Coronel, región de Biobio, Chile. Subsecretaria del Medio Ambiente SEREMI BIOBIO.

8. García-Méndez A, Lorenzo C, Vazquez LB, Reyna-Hurtado R (2014) Roedores y murciélagos en espacios verdes en San Cristóbal de Las Casas, Chiapas, México. Therya 5 (2): 615-632. <https://doi.org/10.12933/therya-14-207>

9. González Castaño LA, Buitrago Ramírez MI (2014) Caracterización biológica del humedal Siracusa, como propuesta para su declaratoria como suelo de protección por parte del municipio de Sevilla-Valle del Cauca. Undergraduate Thesis, Universidad del Quindío.

10. Iannacone J, Atasi M, Bocanegra T, Camacho M, Montes A, Santos S, Zuñiga H, Alayo M (2010) Diversidad de aves en el humedal Pantanos de Villa, Lima, Perú: periodo 2004-2007. Biota Neotropica 10 (2): 295-304. <https://doi.org/10.1590/S1676-06032010000200031>

11. Jerez-Ramírez NA, Arriaga-Weiss SL, Ruiz-Campos G, Gama-Campillo LM, Salcedo-Mesa MA, Villanueva-García C, Mata-Zayas EE, Valdez-Leal JD (2023) Composición y diversidad espaciotemporal de la comunidad de aves acuáticas en la laguna de las Ilusiones, Tabasco, México. Ciencias Marinas 49: e3355. <https://doi.org/10.7773/cm.y2023.3355>

12. Jocou AI, Gandullo R (2020) Diversidad de plantas vasculares de los humedales de la Norpatagonia (Argentina). Revista del Museo Argentino de Ciencias Naturales 22 (2): 131-154. <https://dx.doi.org/10.22179/revmacn.22.688>

13. josens ML, Escalante AH, Favero M (2012) Diversity, seasonality and structure of bird assemblages associated with three wetlands in the Southeastern Pampas, Argentina. Ardeola 59 (1): 93-190. <https://dx.doi.org/10.13157/arla.59.1.2012.93>

14. Kusch A, Cárcamo J, Gómez H (2008) Aves acuáticas en el humedal urbano de Tres Puentes, Punta Arenas (53° S), Chile Austral. Anales Instituto Patagonia (Chile) 36 (2): 45-51. <http://dx.doi.org/10.4067/S0718-686X2008000200005>

15. Llamazares Vegh S, Villatarco Vázquez AP, Kunert MC, Tombari AD (2012) Diversidad de vertebrados acuáticos de tres humedales urbanos de la ciudad de Buenos Aires. Biología Acuática 27: 149-161.

16. Machado NG, Rocha LA, das Dores Silva N, da Silva DFN, Florêncio FP, Fernandes TBS (2017) Biodiversity in urban green space: A case study in the Neotropics. Nativa 5 (5): 320-329. <https://doi.org/10.31413/nativa.v5i5.4466>

17. Manrriquez-Gomez FJ, González-Gutiérrez NS, Ortiz-Serrato L, Moreno-Higareda HR, Valdez-Villavicencio JH (2021) Anfibios y reptiles del estero de Punta Banda, Ensenada, Baja California, México. Revista Latinoamericana de Herpetología 4 (2): 74-84. <https://doi.org/10.22201/fc.25942158e.2021.02.247>

18. Menin M, Ferreira RFB, Melo IB, Gordo M, Hattori GY, Sant'Anna BS (2019) Anuran diversity in urban and rural zones of the Itacoatiara municipality, central Amazonia, Brazil. Acta Amazonica 49 (2): 122-130. <https://doi.org/10.1590/1809-4392201800284>

19. Montoya-Osorio J, Arango-Lopera A, García-Arias E, Isaza-Villa S (2023) Composición, riqueza y abundancia de aves acuáticas del lago Tulio Ospina, municipio de Bello, departamento de Antioquia. Boletín SAO 32 (1&2): 24-34.

20. Oro N, Moser CF, Dalzochio MS, de Oliveira MZ, Hadi A, Preuss JF, Tozetti AM (2024) Landscape use and habitat configuration effects on amphibian diversity in southern Brazil wetlands. Wetlands 44: 12. <https://doi.org/10.1007/s13157-023-01766-4>

21. Pacheco V, Zevallos A, Cervantes K, Pacheco J, Salvador J (2015) Mamíferos del refugio de vida silvestre los Pantanos de Villa, Lima-Perú. Científica 12 (1): 26-41. <https://doi.org/10.21142/cient.v12i1.163>

22. Pereyra LC, Akmentins MS, Salica MJ, Quiroga MF, Moreno CE, Vaira M (2021) Tolerant and avoiders in an urban landscape: anuran species richness and functional groups responses in the Yungas’ forest of NW Argentina. Urban Ecosystems 24: 141-152. <https://doi.org/10.1007/s11252-020-01025-y>

23. Rivas Mogollón EL, Pariapaza Liviapoma E, Nuñez Cortez EI (2013) Aves del humedal de Santa Julia, Piura – Perú. Boletín Informativo UNOP 8 (1): 10-20.

24. Rodrigues AG, Borges-Martins M, Zilio F (2018) Bird diversity in an urban ecosystem: the role of local habitats in understanding the effects of urbanization. Iheringia. Série Zoologia 108: e2018017. <https://doi.org/10.1590/1678-4766e2018017>

25. Rojas C, Sepúlveda-Zúñiga E, Barbosa O, Rojas O, Martínez C (2015) Patrones de urbanización en la biodiversidad de humedales urbanos en Concepción metropolitano. Revista de Geografía Norte Grande 61: 181-204. <http://dx.doi.org/10.4067/S0718-34022015000200010>

26. Rojas C, Sepúlveda E, Jorquera F, Munizaga J, Pino J (2022) Accessibility disturbances to the biodiversity of urban wetlands due to built environment. City and Environment Interactions 13: 100076. <https://doi.org/10.1016/j.cacint.2021.100076>

27. Sacco AG, Rui AM, Bergmann FB, Müller SC, Hartz SM (2015) Perda de diversidade taxonômica e funcional de aves em área urbana no sul do Brasil. Iheringia. Série Zoologia 105 (3): 276–287. <https://doi.org/10.1590/1678-476620151053276287>

28. Tapia-Ramírez G, Lorenzo C, Carrillo-Reyes A, Navarrete D, Retana O (2022) Effect of an urban area in the distribution pattern and diversity of Neotropical rodents. Therya 13 (2): 183-193. <https://doi.org/10.12933/therya-22-2100>

29. Tardone RA, Pincheira-Ulbrich JM, Alarcón XX (2024) Inventory of birds in two urban wetlands in Temuco (Chile): A basis for monitoring species. Biota Neotropica 24 (3): e20231585. <https://doi.org/10.1590/16760611-BN-2023-1585>

**Table S4** Information on urban wetlands located in six Neotropical countries, which was extracted from studies referred to in Appendix 1 and compared to the data from this study. NR = not reported

| **Country** | **Name of wetland** | **Total area (ha)** | **Coordinates** | **Taxa studied** | **Ref.** |
| --- | --- | --- | --- | --- | --- |
| Argentina | Laguna Regatas  Laguna Rosedal  Laguna Planetario | 9.97  4.94  0.95 | -34.557, -58.434  -34.572, -58.415  -34.569, -58.411 | Birds  Mammals  Reptiles | 15 |
| Argentina | Laguna Los Padres | 216 | -37.943, -57.724 | Birds | 13 |
| Argentina | Laguna Maturana  Lagunas de Plottier  Laguna San Lorenzo  Laguna natural ribereña Balsa Las Perlas  Laguna natural ribereña La Herradura | 15  1.16  15  1  0.5 | -39.012, -68.438  -38.963, -68.331  -38.945, -68.132  -38.980, -68.123  -38.969, -68.187 | Plants | 12 |
| Argentina | San Salvador de Jujuy ^a^  (29 ponds) | ~0.15 | -24.198, -65.286 | Amphibians | 22 |
| Brazil | Canoas ^a^  (118 sites) | NR | -29.921, -51.180 | Birds ^b^ | 24 |
| Brazil | Cuiabá ^a^ | 33 | -15.663, -55.989 | Plants  Mammals  Reptiles | 16 |
| Brazil | Itacoatiara ^a^  (10 urban sites) | NR | -3.134, -58.434 | Amphibians | 18 |
| Brazil | Pelotas ^a^  (216 point counts) | NR | -31.764, -52.320 | Birds | 27 |
| Brazil | Rio Sinos  (10 ponds) | ~8 | -29.810, -51.157  -29.677, -50.780 | Amphibians | 20 |
| Chile | Boca Maule | NR | -36.998, -73.183 | Amphibians  Mammals  Reptiles | 7 |
| Chile | Laguna Verde  Lenga  Cuatro Esquinas  Rocuant-Andalién  San Andrés  Paicaví | 9.9  480.4  24.3  1183.4  44.9  24.9 | -36.793, -73.160  -36.771, -73.150  -36.775, -73.116  -36.747, -73.076  -36.788, -73.079  -36.803, -73.073 | Plants | 25 |
| Chile | Los Batros | 133 | -36.836, -73.124 | Plants | 26 |
| Chile | Tres Puentes | NR | -53.117, -70.879 | Birds | 14 |
| Chile | Vegas de Chivilcán | 450 | -38.714, -72.616 | Birds | 29 |
| Colombia | Bogotá ^a^  (12 locations) | NR | 4.662, -74.101 | Birds ^c^ | 6 |
| Colombia | El Salitre | 6.4 | 4.667, -74.088 | Plants | 3 |
| Colombia | Lago Tulio Ospina | 4 | 6.328, -75.548 | Birds | 19 |
| Colombia | Siracusa | 0.061 | 4.269, -75.931 | Plants  Amphibians  Mammals  Reptiles | 9 |
| México | Estero de Punta Banda ^d^ | 2360 | 31.737, -116.637 | Amphibians  Reptiles | 17 |
| México | Laguna de las Ilusiones | 259.2 | 18.008, -92.931 | Birds | 11 |
| México | Puerto Interior Turístico Jocotepec (Lago de Chapala) | 9.3584 | 20.284, -103.414 | Birds | 5 |
| México | María Eugenia  La Kisst  Rancho Nuevo | 116  110  3 | 16.713, -92.6213  16.730, -92.651  16.669, -92.572 | Plants | 4 |
| México | El Cerrito  La Kisst  Na-Bolom  La Hormiga  La Albarrada  Los Humedales  Navajuelos | 51.83  53.06  67.76  68.18  39.24  34.45  35.39 | 16.731, -92.646  16.730, -92.651  16.742, -92.630  16.742, -92.624  16.711, -92.627  16.708, -92.614  16.713, -92.616 | Plants  Mammals | 8 |
| México | Albarrada-Minas  Cerrito SEDEM | NR | 16.710, -92.625  16.731, -92.646 | Mammals | 28 |
| Perú | Chimbote ^a^  (2 zones) | NR | -9.099, -78.546 | Plants | 1 |
| Perú | Pantanos de Villa | 263 | -12.215, -76.984 | Amphibians  Reptiles | 2 |
| Perú | Pantanos de Villa:  Laguna Génesis  Laguna Mayor  Laguna Marvilla | 0.21  5  0.336 | -12.211, -76.987  -12.207, -76.991  -12.223, -76.989 | Birds | 10 |
| Perú | Pantanos de Villa | 263.27 | -12.212, -76.990 | Mammals | 21 |
| Perú | Santa Julia | 12 | -5.202, -80.664 | Birds | 23 |
| Costa Rica | Laguna Doña Ana  Recinto Paraíso | 9  4 | 9.833, -83.878  9.827, -83.869 | Plants  Amphibians  Birds  Mammals  Reptiles | This study |

^a^ Name of the city or municipality where the wetland(s) is(are) located.

^b^ We only considered the species whose habitat was classified by the authors as wetland, marsh, water, or urban.

^c^ We only considered the species classified by the authors as utilizers and dwellers of urban habitats.

^d^ Sub-estuarine wetland.


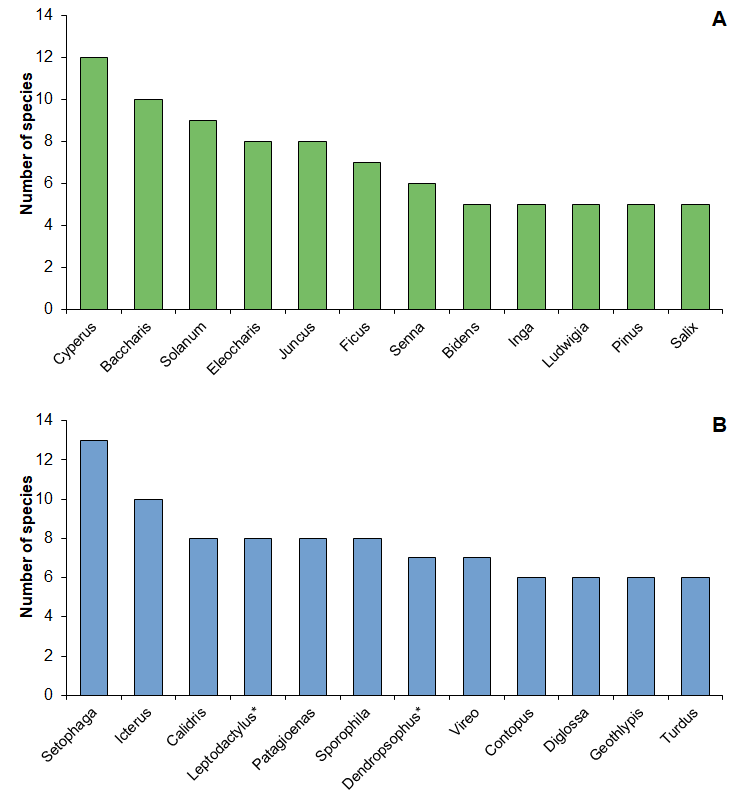


**Fig. S3** Genera of plants (A) and terrestrial vertebrates (B) with the highest number of species reported in the studies from Appendix 1. All genera of animals are birds except for those with an asterisk (*)
